# Rapid and Non-Destructive Assessment of Eight Essential Amino Acids in Foxtail Millet: Development of an Efficient and Accurate Detection Model Based on Near-Infrared Hyperspectral

**DOI:** 10.3390/foods14213760

**Published:** 2025-11-01

**Authors:** Anqi Gao, Xiaofu Wang, Erhu Guo, Dongxu Zhang, Kai Cheng, Xiaoguang Yan, Guoliang Wang, Aiying Zhang

**Affiliations:** 1 Shanxi Houji Laboratory, Taiyuan 030031, China; gaq1137804400@163.com (A.G.); guoerhuo2003@163.com (E.G.); 2 Institute of Millet Research, Shanxi Agricultural University, Changzhi 046011, China; gzszdx@163.com (D.Z.); kaikai6622@126.com (K.C.); gzsyxg001@163.com (X.Y.); 3 Department of Mechanical Engineering, Shanxi Institute of Engineering Technology, Yangquan 045000, China; 4 Department of Scientific Research Management, Shanxi Agricultural University, Taigu 030801, China; 19935150928@163.com

**Keywords:** millet, essential amino acids, near-infrared hyperspectral technology, machine learning models, grain quality evaluation

## Abstract

Foxtail millet is a vital grain whose amino acid content affects nutritional quality. Traditional detection methods are destructive, time-consuming, and inefficient. This work established a rapid and non-destructive method for detecting essential amino acids in the foxtail millet. To address these limitations, this study developed a rapid, non-destructive approach for quantifying eight essential amino acids—lysine, phenylalanine, methionine, threonine, isoleucine, leucine, valine, and histidine—in foxtail millet (variety: Changnong No. 47) using near-infrared hyperspectral imaging. A total of 217 samples were collected and used for model development. The spectral data were preprocessed using Savitzky–Golay, adaptive iteratively reweighted penalized least squares, and standard normal variate. The key wavelengths were extracted using the competitive adaptive reweighted sampling algorithm, and four regression models—Partial Least Squares Regression (PLSR), Support Vector Regression (SVR), Convolutional Neural Network (CNN), and Bidirectional Long Short-Term Memory (BiLSTM)—were constructed. The results showed that the key wavelengths selected by CARS account for only 2.03–4.73% of the full spectrum. BiLSTM was most suitable for modeling lysine (R^2^ = 0.5862, RMSE = 0.0081, RPD = 1.6417). CNN demonstrated the best performance for phenylalanine, methionine, isoleucine, and leucine. SVR was most effective for predicting threonine (R^2^ = 0.8037, RMSE = 0.0090, RPD = 2.2570), valine, and histidine. This study offers an effective novel approach for intelligent quality assessment of grains.

## 1. Introduction

Foxtail millet (*Setaria italica*), a traditional advantageous coarse cereal originating from China, has a cultivation history spanning thousands of years [1]. Thanks to its drought resistance and tolerance to poor soils, it is widely cultivated in regions such as the Loess Plateau and the North China Plain, serving as a crucial crop for ensuring food security in arid and semi-arid areas. Simultaneously, the foxtail millet represents a key source of “whole grain nutrition” in the human dietary structure [2]. Its protein content reaches approximately 15%, significantly higher than that of staple grains such as rice and corn. It is also rich in dietary fiber, B vitamins, and trace nutrients like iron and zinc, making it recognized as a key future food crop [2,3]. With growing emphasis on healthy eating, the foxtail millet has experienced sustained growth in market demand due to its balanced nutrition and easy digestibility [4,5,6,7]. In 2024, China’s annual foxtail millet production exceeded 20 million metric tons, making it one of the most valuable categories within the coarse cereal industry. The core nutritional value of foxtail millet lies in its amino acid composition—it contains all eight essential amino acids that cannot be synthesized by the human body and must be obtained from food. These amino acids serve as fundamental building blocks for maintaining physiological functions. Lysine (Lys), the primary limiting amino acid, is crucial for child development [8,9,10,11,12]. Phenylalanine (Phe) serves as a precursor for neurotransmitters, while methionine (Met) drives methylation reactions and antioxidant synthesis [9,10]. Threonine (Thr) maintains mucosal integrity and immune function [10,11]. The branched-chain amino acids—isoleucine (Ile), leucine (Leu), and valine (Val)—regulate muscle metabolism and energy homeostasis [13]. Histamine (His) mediates immune responses, regulates inflammation, and stimulates gastric acid secretion [14]. Accurate quantification of these eight essential amino acids in foxtail millet provides critical data for nutritional evaluation, variety improvement, and processing optimization, advancing nutrition-oriented agriculture.

However, current methods for detecting amino acids in foxtail millet primarily rely on traditional chemical analysis. This procedure involves multiple steps: grinding, sieving, extraction, acid hydrolysis, and filtration, followed by detection using an amino acid autoanalyzer [8,10,12,15]. Although this method delivers high detection accuracy, it presents several significant limitations. First, the process is tedious and time-consuming, often requiring several days for completion, making it unsuitable for applications demanding high-throughput rapid analysis (e.g., breeding screening) or real-time monitoring during processing operations. Second, the method is destructive: the acid hydrolysis irreversibly alters the sample structure, preventing sample reuse and increasing detection costs. Furthermore, it poses environmental and safety concerns—the concentrated hydrochloric acid used is highly corrosive, jeopardizing operator safety and generating hazardous waste that contaminates the environment. These limitations render traditional methods incompatible with modern coarse grain industry requirements for efficiency, sustainability, and real-time monitoring. Consequently, the developing novel nondestructive detection techniques have emerged as a pressing need for the industry.

The emergence and advancement of near-infrared hyperspectral technology have pioneered a novel pathway and provided a practical technical solution for high-throughput rapid detection of key nutritional components in agricultural products—particularly complex systems such as multiple essential amino acids in millet. This technology is based on the overtone and combination band absorption effects generated by molecular vibrations in substances. It enables the simultaneous acquisition of both spectral information and spatial distribution characteristics of samples, forming a high-resolution “spectral fingerprint” that comprehensively captures compositional data [16]. Compared to traditional detection methods, this technology offers significant advantages, including non-destructive analysis, rapid detection, simultaneous multi-component measurement, and elimination of chemical pollution. It demonstrates broad application prospects in the fields of agricultural product quality analysis and intelligent detection. The different amino acid molecules contain functional groups such as N–H, C–H, and O–H bonds [17,18,19], which exhibit distinct absorption behaviors in the near-infrared region, forming characteristic absorption peaks closely correlated with their concentrations. By leveraging chemometric methods and data analysis algorithms, these characteristic features can be extracted from complex spectra. A quantitative calibration model can be established between the spectral signals and target compound concentrations, enabling rapid quantitative analysis without sample pretreatment, non-contact and non-destructive manner. This approach significantly enhances detection efficiency and accuracy while maximally preserving the original state and integrity of the samples. In recent years, the near-infrared hyperspectral technology has achieved remarkable results in agricultural product quality inspection, with continuously expanding applications. Yang [20] combined near-infrared spectroscopy with chemometrics and deep learning, using Competitive Adaptive Reweighted Sampling to optimize feature wavenumber reduction and minimize redundancy. A corn quality classification and detection model was constructed based on LeNet-5. Shu [21] utilized hyperspectral imaging technology to estimate the amino acid content in corn leaves, employing Partial Least Squares Regression (PLSR) to develop prediction models for β-aminobutyric acid, ornithine, citrulline, Met, and His. This approach contributes to corn yield estimation and enhances nitrogen use efficiency. Fei [22] applied hyperspectral technology to detect the protein and fat content in sorghum, using three different combined algorithms to extract feature wavelengths and establishing regression models with Random Forest, Backpropagation-Genetic Algorithm, Support Vector Regression, and PLSR. Despite significant progress in quality detection of various crops using this technology, systematic research on essential amino acids in foxtail millet remains relatively limited. The complex chemical composition of foxtail millet, diverse types of amino acids, and significant variations in their concentrations collectively pose substantial challenges for rapid and non-destructive detection. Currently, there is a lack of systematic modeling research on the near-infrared hyperspectral detection of multiple essential amino acids in millet. Furthermore, the accuracy, robustness, and applicability of existing models require further improvement. Therefore, systematically advancing research on the application of near-infrared hyperspectral technology in millet amino acid detection is of significant practical importance and has far-reaching implications for improving the millet quality evaluation system and promoting high-quality industry development.

This study focuses on foxtail millet from the high-quality production area in the eastern Loess Plateau of Shanxi Province. A rapid non-destructive detection model for eight essential amino acids was developed using near-infrared spectroscopy. An integrated preprocessing approach was applied to enhance data quality, followed by the use of a feature wavelength selection algorithm to extract key wavelengths related to the amino acids. Four machine learning-based prediction models were ultimately constructed. By evaluating model performance metrics in combination with the characteristics of the amino acids, an optimal model selection mechanism was established, resulting in an efficient, accurate, and cost-effective detection solution. This methodology provides technical support for nutritional quality screening in foxtail millet breeding, monitoring nutrient retention during processing, and market-based quality grading. It also serves as a reference for amino acid testing in other coarse grains such as oats and buckwheat, thereby promoting the standardization and industrial application of whole-grain nutritional evaluation technologies.

## 2. Materials and Methods

### 2.1. Collection of Foxtail Millet Samples

The study was performed at the breeding base of the Institute of Millet Research, Shanxi Agricultural University (36°12′ N, 113°08′ E). The region has a stable annual average temperature of 10.2 °C, a frost-free period of 185 days, an annual precipitation of approximately 550 mm, and an elevation of 977 m at the base, fully meeting the thermal requirements for the complete growth cycle of foxtail millet. The experimental field features typical cinnamon soil with strong water and nutrient retention capacity, moderate organic matter content, and good suitability for foxtail millet root development and nutrient uptake. Stanley compound fertilizer (N-P_2_O_5_-K_2_O = 25-10-16, Stanley Agricultural Group Inc., Shandong, China) was applied as base fertilizer. This nutrient ratio provided balanced N, P, and K nutrition during the millet seedling stage, promoting robust early growth. Manual sowing of foxtail millet variety Changnong No.47 was completed on 10 May 2024, using the dibbling method with row spacing of 30 cm and plant spacing of 10 cm. At the jointing stage, urea was top-dressed at a rate of 225 kg/hm^2^ through the drip irrigation system, enhancing fertilizer use efficiency via integrated water-fertilizer management. Centralized harvesting was conducted on 5 October 2024, when the foxtail millet grains had fully matured. To ensure sample representativeness, a five-point random sampling method was employed within the foxtail millet cultivation plots, evenly covering different growth areas. This approach yielded 217 samples, each weighing 200 g, meeting the requirements for both sample quantity and randomness in subsequent analyses. After harvesting, the samples were first sun-dried naturally until the moisture content dropped below 13%, followed by mechanical dehulling to obtain clean foxtail millet grains.

### 2.2. Hyperspectral Image Acquisition and Data Extraction

The near-infrared hyperspectral data of foxtail millet samples were collected using a push-broom hyperspectral imaging system (Headwall Photonics, Hexagon AB, MA, USA). The system was configured with the following parameters: spectral range of 900–1700 nm (172 spectral bands; spectral resolution: 4.715 nm), object-to-image distance of 280 mm, and scan speed of 2.721 mm/s. Prior to data acquisition, a rigorous calibration procedure was performed. This included blank reference calibration using a standard diffuse reflection panel and dark reference calibration with the lens covered to eliminate sensor noise. All calibrations were repeated before each batch of samples to ensure data accuracy. The calibration was according to Equation (1):

(1) $$R=\frac{R_{0}-R_{b}}{R_{w}-R_{b}}$$

where *R* is the corrected image, *R*_0_ represents the original raw image, *R_w_* is the blank reference calibration image (reflectivity ≈ 99.9%) and *R_b_* is the dark reference calibration image (reflectivity ≈ 0%).

The foxtail millet samples were evenly filled into a container measuring 3 cm in diameter and 1 cm in depth, with the surface leveled and compacted. Each sample underwent three consecutive spectral data acquisitions. Given the system’s significantly reduced signal-to-noise ratio at the edge regions of the spectral response range (<950 nm and >1650 nm), we selected the 950–1650 nm spectral range for modeling, ensuring both the stability of the spectral data and the validity of the extracted features. This effective spectral band comprises 148 characteristic wavelengths and is well-suited for constructing and analyzing hyperspectral quantitative detection models for the content of eight major amino acids in foxtail millet.

To efficiently select regions of interest (ROI) and achieve batch extraction and processing of spectral data, we designed a batch processing program for hyperspectral data by performing secondary development of SpectralView software III (Headwall Photonics, Hexagon AB, Boston, MA, USA) based on Visual Basic 6.0 (Microsoft, Washington, DC, USA). The program comprises two core modules: an ROI coordinate generation module and a batch data processing module. Using an elliptical model, we defined the center coordinates, semi-axis lengths, and axial intervals for each ROI. All pixels within the ROIs were systematically captured following a left-to-right, top-to-bottom scanning sequence, generating a corresponding coordinate matrix. After importing the hyperspectral image into the SpectralView software, the program automatically extracts the reflectance data within each ROI based on this coordinate matrix. The batch processing module further performs mean calculation on the extracted spectral data and outputs the average reflectance results. A total of 7200 pixels (i.e., 2400 × 3) were collected for each millet sample, and the average spectrum of each sample was used as the foundational dataset for subsequent data analysis.

### 2.3. Amino Acid Content Measurement

The millet samples that had undergone spectral collection were ground and passed through a 60–mesh sieve. Then, 0.1 g of the millet powder was weighed, mixed with 10 mL of 6 mol/L HCl, and purged with nitrogen for 3 min. The mixture was placed in a constant-temperature oven at 110 °C for 24 h, filtered, and diluted to a final volume of 25 mL. A 2 mL aliquot of the sample solution was taken and evaporated to dryness using a rotary evaporator at 55 °C. Next, 0.5 mL of pure water was added twice, and the solution was evaporated to dryness each time. After that, 1 mL of pure water was added and again evaporated to dryness. Finally, 4 mL of solution was added, and the mixture was shaken thoroughly. The solution was drawn using a 1 mL syringe, filtered through a 0.45 μm filter into an injection vial, and analyzed using an amino acid autoanalyzer (LC-2300 AAA, Beijing Huaneng Times Technology Co., Ltd., Beijing, China) [7,8,9,10,11,12,15,23,24].

### 2.4. Data Preprocessing and Dataset Partitioning

During the acquisition of spectral images by the hyperspectral imaging system, factors such as instrumental noise, environmental interference, and surface scattering inevitably introduce artifacts into the experimental data. Therefore, preprocessing of the raw spectral data is critical to eliminate the effects of instrumental and environmental noise, suppress surface scattering, and effectively reduce interference in the spectral signals.

The Savitzky–Golay (S–G) [25,26] smoothing is a nonlinear filtering method based on local polynomial fitting, primarily employed to eliminate high-frequency noise from spectral curves. This technique performs least-squares fitting using a polynomial function within a predefined local window of specified width, enabling effective noise reduction through spectral smoothing. In this study, the parameters were configured as follows: polynomial order = 1, frame length = 3, and window size = 5 using a finite impulse response smoothing filter.

The adaptive iteratively reweighted penalized least squares (airPLS) [27,28] is a baseline correction method used for spectral data preprocessing, designed to remove baseline drift interference and enhance data accuracy. The algorithm operates by iteratively constructing an asymmetric weight matrix to distinguish between meaningful spectral signals and baseline components. Through weighted least-squares fitting, it derives an optimal baseline, achieving clear separation between the original spectral data and the estimated baseline. The parameter settings in this study were: smoothing penalty parameter = 10^4^, differential matrix order = 2, weight update exponent = 0.1, negative deviation ratio = 0.05, and iteration count = 800.

The standard normal variate (SNV) [29,30] is a single-sample statistical correction method that eliminates multiplicative scattering and background interference in reflectance spectra caused by variations in particle size, surface gloss, and sample distribution. This technique processes individual spectral samples through sequential centralization and standardization, resulting in a data distribution that approximates a standard normal distribution and effectively suppresses the specified interference effects.

During model development and validation, the hold-out method was implemented to randomly divide the preprocessed sample set into training and prediction subsets at a 3:1 ratio, thereby ensuring model generalizability and predictive reliability. The training set facilitated model training, parameter optimization, and structural adjustments to capture inherent data patterns, while the prediction set—maintained completely independent from the training procedure—was used to objectively evaluate the model’s predictive performance on unknown samples.

### 2.5. Introduction to Key Wavelength Extraction Algorithm

The competitive adaptive reweighted sampling (CARS) [31,32,33,34] algorithm mimics the principle of “survival of the fittest” from Darwinian evolution to identify optimal combinations of informative spectral variables. The CARS employs the following steps to screen key wavelengths:

Step 1: Using Monte Carlo Sampling (MCS), 80% of the training set samples were randomly selected to establish a PLSR model, ensuring stability and generalization capability under varying sampling conditions. The regression coefficient |*k_i_*| for the *i*-th wavelength (*i* = 1, 2,…, *p*) was obtained.

Step 2: An Exponentially Decreasing Function (EDF) is applied to eliminate wavelengths with small |*k_ᵢ_*| values. The variable retention rate is defined as *r_ⱼ_* = ae^−^*ᵇʲ* (*j* = 1, 2,…, *N*), where *j* denotes the *j*-th Monte Carlo sampling iteration. *N* represents the total number of MCS iterations. Parameters a and b are constants calculated based on *r*_1_ = 1 and *r_N_* = 2/*p*, using the following formulas:

(2) $$a=(p/2{)}^{1/(N-1)}$$

(3) b=ln(p/2)/(N−1)

Step 3: Further variable screening is performed using Adaptive Reweighted Sampling (ARS) technology. Mimicking the “survival of the fittest” principle in Darwinian evolution, variables are selected based on their weights evaluated by the following formula:

(4) $$w_{i}=\left|k_{i}\right|/\sum_{i=1}^{p}\left|k_{i}\right|$$

Step 4: The above steps are repeated until the number of MCS iterations reaches the predefined value *N* (set to 500 in this study).

Step 5: The Root Mean Square Error of Cross–Validation (RMSECV) value obtained from 10-fold cross-validation is used as the evaluation criterion. The variable subsets generated from each MCS iteration are compared, and the subset corresponding to the minimum RMSECV value is selected as the optimal variable combination.

### 2.6. Synchronous Detection Model Construction

The partial least squares regression (PLSR) [20,21,22,35,36,37] is a multivariate statistical method based on principal component analysis. By performing principal component decomposition on both the spectral matrix and the physicochemical parameter matrix, it extracts components with the highest covariance between the two, effectively reducing data dimensionality and enhancing predictive capability. PLSR determines the optimal number of principal components through cross-validation to prevent overfitting or underfitting. The performance of the model was evaluated using the Root Mean Square Error (RMSE), ultimately establishing a robust linear relationship between spectral and physicochemical parameters to achieve accurate prediction of physiological parameters. This model effectively captures the linear relationships between spectral features and target parameters, demonstrating strong interpretability and predictive power.

The support vector regression (SVR) [35,36,37] is a machine learning method based on statistical learning theory. Its core principle involves using an *ε*-insensitive loss function to control error tolerance and identifying an optimal hyperplane in the feature space to maximize the model’s generalization capability. SVR employs kernel functions to map data into a high-dimensional feature space, transforming the problem into a linear regression task and thereby effectively capturing complex relationships. Through cross-validation optimization of the penalty coefficient *C*, *ε*, and kernel parameters, SVR can avoid overfitting. Ultimately, a robust predictive model is established. It demonstrates superior accuracy and generalization performance compared to traditional methods under high-dimensional, small-sample scenarios.

The convolutional neural network (CNN) [34,35,36,37] is a deep learning method designed for processing grid-structured data. It automatically extracts local features through convolutional layers, enhances robustness and reduces dimensionality via pooling layers, and integrates high-level features through fully connected layers to achieve end-to-end regression prediction. CNN regression can autonomously uncover complex nonlinear relationships within data without requiring manual feature engineering. Leveraging regularization, Dropout, and cross-validation to optimize hyperparameters, the model effectively controls overfitting while demonstrating excellent generalization capability. Ultimately, achieving high-precision prediction of continuous variables. This study employed the Adam optimization algorithm with a maximum of 5000 training epochs, a gradient threshold of 1, and an initial learning rate of 0.01. The piecewise learning rate decay strategy was applied, reducing the rate every 100 epochs with a decay factor of 0.2. The L2 regularization coefficient of 0.01 was used to mitigate overfitting.

The bidirectional long short-term memory (BiLSTM) [36,37,38,39,40] is a deep learning method designed for sequential data. Its core strength lies in its bidirectional architecture and gating mechanisms. The forward and backward LSTMs extract features from both directions, effectively capturing long-term dependencies and integrating contextual information. This enables a comprehensive understanding of sequence dynamics. This approach effectively prevents overfitting through regularization, Dropout, and hyperparameter optimization, while automatically uncovering nonlinear temporal relationships within the data. Ultimately, achieving high accuracy and generalization capability prediction of continuous variables.

Both the key wavelength extraction and model construction algorithms were executed for 50 independent runs, respectively. The predictive performance of the models was evaluated using the coefficient determination (R^2^), RMSE, and relative percent deviation (RPD), and the optimal result was selected. The closer R^2^ is to 1, the lower the RMSE value, and the higher the predictive accuracy of the model. Meanwhile, when RPD > 2, it indicates that the model achieves a good predictive effect for the indicator. When 1.4 < RPD < 2, it means the model can predict the indicator to a certain extent. When RPD < 1.4, the model is unable to predict the indicator [32]. MATLAB software (Version 2023b, MathWorks, Natick, MA, USA) was used for data processing and analysis.

## 3. Results and Analysis

### 3.1. Amino Acid Content and Correlation Analysis of Foxtail Millet

**Figure 1:**
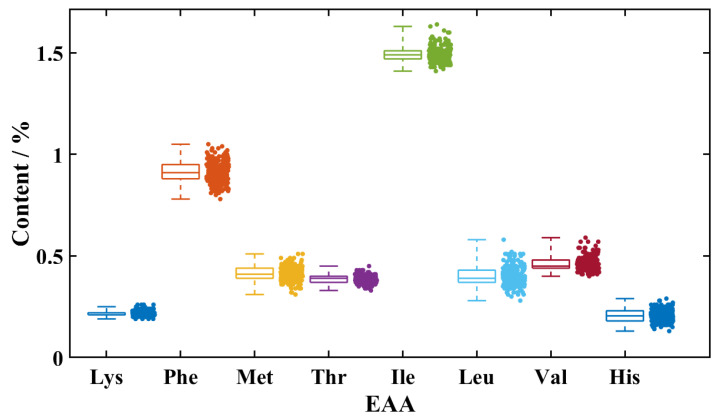
Quantification results of eight EAA in 217 Foxtail Millet Samples. (The abbreviations represent: Lys (Lysine), Phe (Phenylalanine), Met (Methionine), Thr (Threonine), Ile (Isoleucine), Leu (Leucine), Val (Valine), and His (Histidine). The box plots illustrate the distribution characteristics of each amino acid content. The adjacent scatter points represent the corresponding amino acid concentrations from the 217 foxtail millet samples.

As an important coarse grain, foxtail millet has a protein content as high as 11.53%, offering significant nutritional advantages. Figure 1 shows the distribution characteristics of eight essential amino acids (EAA) in foxtail millet. The Shapiro–Wilk test confirmed that all amino acid data conformed to normal distribution, and boxplot analysis showed no outliers. These distribution characteristics indicate reliable data quality, making the dataset suitable for subsequent statistical modeling and analysis. Amino acid composition analysis of proteins showed that foxtail millet contains eight essential amino acids for humans, including Lys, Phe, Met, Thr, Ile, Leu, Val, and His. These essential amino acids accounted for 38.94% of the total protein content in the studied millet variety. In terms of specific composition, the proportions of different essential amino acids in protein show significant differences. Ile exhibited the highest content at 12.94%, followed by Phe (7.918%), Val (4.001%), Met (3.582%), Leu (3.467%), Thr (3.354%), Lys (1.892%), and His (1.787%). The high content of Ile distinguishes foxtail millet from other grains. This may be associated with its unique metabolic pathways or physiological functions [41,42,43].

### 3.2. Spectral Response and Preprocessing of Foxtail Millet

**Figure 2:**
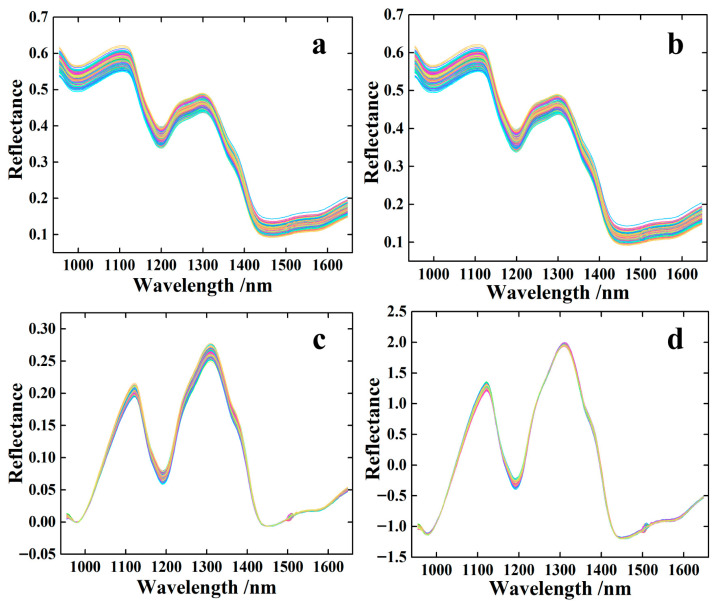
Results of near-infrared spectra and spectral pretreatment for foxtail millet. (**a**) Raw spectral data; (**b**) S–G pretreatment; (**c**) S–G–airPLS pretreatment; (**d**) S–G–airPLS–SNV pretreatment.

Figure 2a shows that the near-infrared spectra of foxtail millet in different wavelength ranges exhibited significant differences. A decreasing trend was observed at 950–1000 nm, 1100–1200 nm, and 1300–1450 nm. This indicated that the absorption of near-infrared light by foxtail millet in these ranges gradually increases. This phenomenon may be related to characteristic absorption of specific chemical components (e.g., moisture, proteins, carbohydrates, etc.) within these ranges. At 1000–1100 nm, 1200–1300 nm, and 1450–1650 nm, an upward trend was observed. This indicated a diminished absorption of near-infrared light at corresponding wavelengths by foxtail millet.

Figure 2b demonstrates that the S–G method effectively suppresses high-frequency random noise in spectral signals. This noise typically manifested as rapid, irregular microfluctuations in the signal. Crucially, while removing noise, the S–G method maximally preserved the key characteristic information of the spectrum, avoiding peak broadening or distortion that may occur with methods such as simple moving average. The clear effect after airPLS processing can be observed through comparison between Figure 2b,c. The background baseline artifacts in Figure 2b, such as overall tilt, curvature, or slow fluctuations, were excellently corrected in Figure 2c. The corrected spectral curves were essentially straightened and stabilized near the zero baseline. This indicated that non-target signal offsets caused by instruments, sample matrices, or environmental factors have been effectively removed. The spectra after SNV processing are shown in Figure 2d. The overall shift in light intensity and scattering effects caused by differences in the physical state of the samples (particle size, surface roughness) was effectively eliminated or reduced. This enhancement improved the spectral comparability across different samples, enabling analytical models to focus more on chemical composition information rather than physical interference.

### 3.3. Key Wavelength Extraction

**Figure 3:**
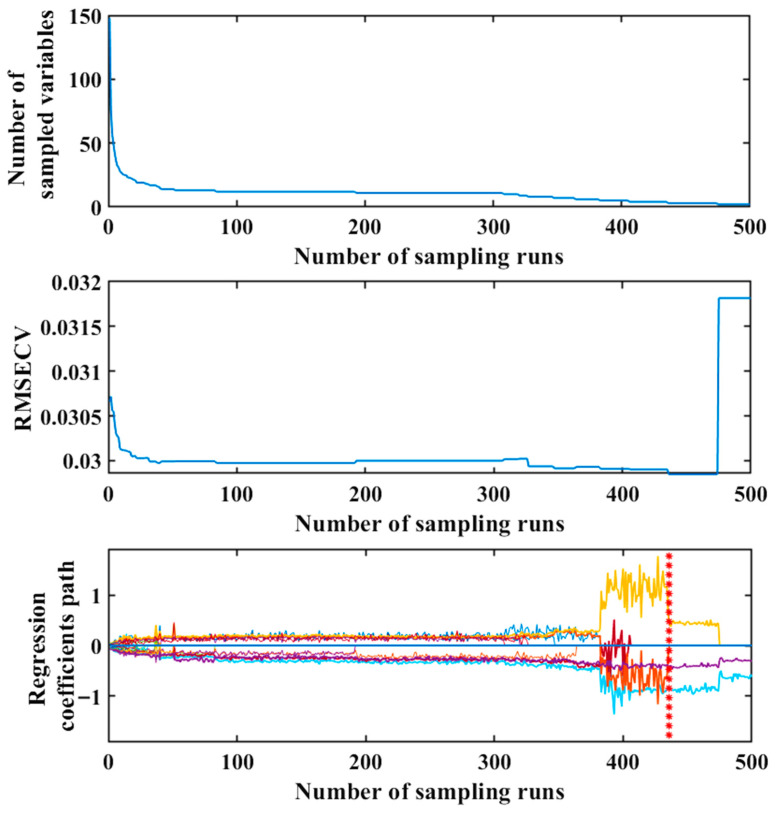
Feature band extraction process for *Ile* using the CARS.

The objective of the CARS is to eliminate irrelevant variables and reduce collinearity among spectral variables. Taking Ile as an example, Figure 3 illustrates the changes in the number of sample variables, RMSECV, and regression coefficient paths within subsets as the number of MCS runs increases. As the number of MCS runs increased, the number of selected variables initially decreased exponentially and then gradually stabilized. The RMSECV values showed a dynamic change, which initially decreased and then increased. This phenomenon indicates that the variables initially removed during the variable screening process were irrelevant to the target component, while subsequently introduced variables in the subset were unrelated to the component of interest. More valid bands were retained at the marked location. At this point, when the sampling count reached 436 runs, the RMSECV achieved its minimum value of 0.0298. The variables selected under these conditions formed the optimal variable set (1143 nm, 1228 nm and 1398 nm). Figures S1–S7 illustrate the process for Lys, Phe, Met, Thr, Leu, Val and His in foxtail millet, respectively. Table 1 lists the key wavelengths extracted for the optimal results of each amino acid.

### 3.4. Construction of Simultaneous Detection Models

**Figure 4:**
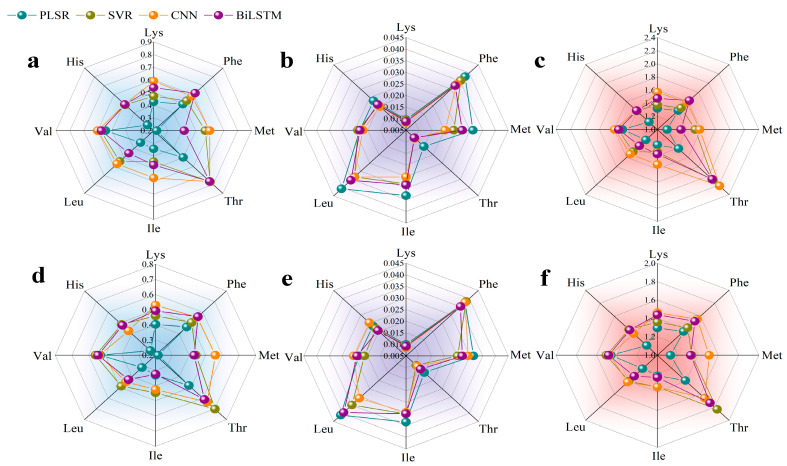
Evaluation results of models established based on key wavelengths for eight essential amino acids in millet. (**a**) R^2^ in the training set; (**b**) RMSE in the training set; (**c**) RPD in the training set; (**d**) R^2^ in the prediction set; (**e**) RMSE in the prediction set; (**f**) RPD in the prediction set.

**Figure 5:**
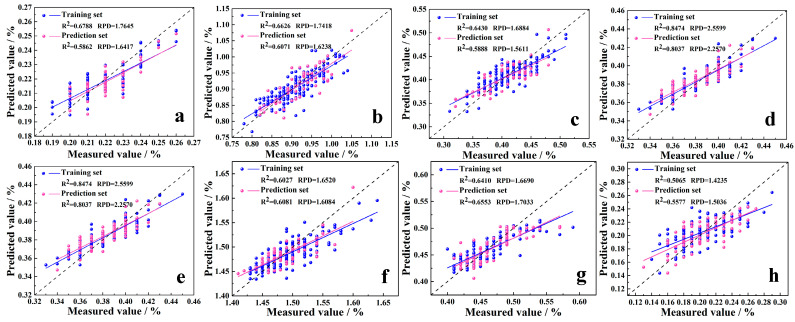
Fitting results of the optimal model established for eight essential amino acids in millet based on key wavelengths. (**a**–**h**) represent Lys, Phe, Met, Thr, Ile, Leu, Val, and His, respectively.

Simultaneous detection models for the content of eight essential amino acids were established and evaluated using both the preprocessed full-spectrum data and the key wavelengths of foxtail millet. The assessment metrics are shown in Figure 4 and Figure S8. Figure S8 illustrates the accuracy of different models trained on the full-spectrum dataset of the eight essential amino acids. Figure 4 demonstrates the accuracy of models trained on the key wavelength dataset. For Lys, PLSR, SVR, CNN, and BiLSTM models were developed using both full-spectrum and key wavelengths. The results indicate that the models built using key wavelengths consistently outperformed those based on full-spectrum data in terms of accuracy. Among these, the BiLSTM model achieved the highest prediction accuracy (R^2^ = 0.5862, RMSE = 0.0081, RPD = 1.6417). Compared to the second-best performing SVR model, the R^2^ improved by approximately 2.57%, and the RPD increased by about 7.46%. For Phe, the CNN model developed based on the key wavelength dataset achieved the best prediction accuracy (R^2^ = 0.6071, RMSE = 0.0332, RPD = 1.6238). Compared to the second-best performing BiLSTM model, the R^2^ improved by approximately 5.88%, and the RPD increased by approximately 3.04%. For Met, the CNN model developed based on the key wavelength dataset achieved the best prediction accuracy (R^2^ = 0.5888, RMSE = 0.0240, RPD = 1.5611). Compared to the second-best performing SVR model, the R^2^ improved by approximately 4.51%, and the RPD increased by approximately 3.15%. For Thr, the SVR model developed based on the key wavelength dataset achieved the best prediction accuracy (R^2^ = 0.8037, RMSE = 0.0090, RPD = 2.2570). Compared to the second-best performing BiLSTM model, the R^2^ improved by approximately 5.24%, and the RPD increased by approximately 4.92%. For Ile, the CNN model developed using the key wavelength dataset achieved the best prediction accuracy (R^2^ = 0.6081, RMSE = 0.0235, RPD = 1.6084). Compared to the second-best performing BiLSTM model, the R^2^ improved by approximately 26.2%, and the RPD increased by approximately 15.7%. For Leu, the CNN model developed based on the key wavelength dataset achieved the best prediction accuracy (R^2^ = 0.5763, RMSE = 0.0295, RPD = 1.5446). Compared to the second-best performing SVR model, the R^2^ improved by approximately 2.33%, and the RPD increased by approximately 2.08%. For Val, the results indicate that the SVR model developed based on the key wavelength dataset achieved the best prediction accuracy (R^2^ = 0.6553, RMSE = 0.0221, RPD = 1.7033). Compared to the second-best performing CNN model, the R^2^ improved by approximately 5.22%, and the RPD increased by approximately 1.68%. For His, the results demonstrate that the SVR model developed based on the key wavelength dataset achieved the best prediction accuracy (R^2^ = 0.5577, RMSE = 0.0200, RPD = 1.5036). Compared to the second-best performing BiLSTM model, the R^2^ improved by approximately 6.65%, and the RPD increased by approximately 3.60%. Figure 5 shows the fitting results of the optimal model established based on the key wavelength dataset for the content of eight essential amino acids.

In summary, the optimal predictive models for the eight essential amino acids in foxtail millet exhibit significant differences in their adaptability. Lys is best predicted by the BiLSTM model. Phe, Met, Ile, and Leu are more effectively modeled using the CNN model. Thr, Val, and His show optimal results with the SVR model. No single model demonstrates universal superiority for all amino acids, highlighting the need for targeted model selection based on specific amino acid characteristics. Under all modeling approaches, the models constructed using key wavelength data consistently demonstrated higher predictive accuracy. PLSR models performed the poorest (most with R^2^ < 0.5 and RPD < 1.4). SVR, CNN, and BiLSTM emerged as superior models. SVR achieved optimal prediction for threonine. CNN demonstrated broad adaptability for multiple amino acids. BiLSTM excelled only for Lys. All three significantly outperformed traditional PLSR.

## 4. Discussion

This study focused on the near-infrared spectroscopy-based prediction of eight essential amino acids in foxtail millet. The results demonstrated that models using key wavelength datasets generally exhibited superior predictive performance compared to those using full-spectrum data. Moreover, significant differences were observed in the optimal regression models for different amino acids. This phenomenon resulted from the combined effects of the unique characteristics of foxtail millet amino acids, their molecular structures, the properties of the key wavelength datasets, and the compatibility with regression models. Meanwhile, the study demonstrates clear advantages while also revealing areas for improvement.

### 4.1. Linkage Effects of Amino Acid Content and Spectral Response in Foxtail Millet

The variation characteristics of amino acid content in foxtail millet directly determine the intensity and overlap of spectral signals, thereby affecting model prediction performance. In terms of content, the eight essential amino acids collectively accounted for 38.94% of the total protein, though with significant variation in their individual proportions. Ile showed the highest contribution. While Lys and His were the lowest. Amino acids with higher concentrations exhibit stronger spectral responses from their characteristic functional groups (e.g., alkyl C–H bonds). This results in higher signal-to-noise ratios, making it easier for models to capture meaningful information (e.g., the key wavelength model for Ile achieved R^2^ = 0.6081, far surpassing the full-spectrum model’s R^2^ = 0.3309). Conversely, amino acids with exceedingly low concentrations (e.g., Lys and His) exhibit spectral signals that are easily dominated by background interference, such as moisture O–H and carbohydrate C–H vibrations, masking the target signals [15,43,44]. The key wavelengths of different amino acids are consistently concentrated within these characteristic spectral regions, confirming the systematic pattern that amino acid spectral responses are embedded within the overall component response of foxtail millet.

### 4.2. Amino Acid Type Determines Model Compatibility

Differences in the molecular structures of amino acids—particularly their side-chain functional groups—determine whether their spectral features exhibit localized or continuous characteristics, thereby influencing their compatibility with different regression models. Aromatic amino acids (Phe) contain a benzene ring in their side chains. They exhibit strong, localized characteristic absorption peaks at 955 nm (benzene ring C–H bending vibration) and 1280 nm (benzene ring stretching vibration), with high signal distinctiveness [11,15]. CNN excel at extracting localized spatial features through convolutional kernels, enabling precise identification of these discrete strong absorption peaks. Therefore, the CNN model for *Phe* demonstrates the best performance. The sulfur-containing amino acids (Met) have a side chain consisting of a thioether group (–S–CH_3_) [15]. Its S–C vibration signals are weak (at 1082 nm and 1129 nm) and easily masked by protein N–H and carbohydrate C–O signals [15,44,45]. The full-spectrum models were severely affected by noise interference, whereas after key wavelength screening (retaining the effective range of 1082–1544 nm), the CNN was able to integrate these weak yet informative local features. Basic amino acids (Lys and His) contain an amino group (–NH_2_) in their side chain [9,46]. Their absorption peaks (at 1172 nm, 1242 nm, and 1388 nm) superimpose with those of carboxyl groups (–COOH) and imino groups (–NH–) in the molecule, resulting in continuous and correlated spectral features [12,13,14,15,46,47]. BiLSTM captures continuous wavelength dependencies through bidirectional temporal modeling, thus achieving optimal performance for Lys prediction. The side chain of His is an imidazole ring. Although it is alkaline, its low concentration and inversely correlated spectral signals with Phe complicate its detection. SVR is required to fit nonlinear relationships through kernel functions. Branched-chain amino acids (Ile, Leu, and Val) feature multi-branched alkyl side chains. Their distinct local C–H vibration characteristics (at 1143 nm and 1186 nm) make them well-suited for CNN–based modeling [7,10]. However, Val, with its longer alkyl side chain, exhibits weakly nonlinear spectral responses, for which SVR’s nonlinear fitting capability proves superior.

### 4.3. The Core Role of Key Wavelength Datasets in Eliminating Redundancy

While full-spectrum data contains abundant information, it also includes substantial redundant wavelengths (e.g., starch scattering signals unrelated to amino acids) and noise (such as instrumental drift and environmental fluctuations in temperature and humidity). This increases the learning burden and amplifies errors of the model. For example, Ile screening identified 1143 nm, 1228 nm, and 1398 nm as key wavelengths (accounting for 2.03% of the total wavelengths). These wavelengths correspond to the stretching vibrations of branched-chain alkyl C–H bonds, directly reflecting the content of Ile [13,15]. For Thr, CARS screened five key wavelengths (ranging from 1186 nm to 1605 nm). These wavelengths cover the overlapping response regions of N–H (proteins) and O–H (moisture), while effectively eliminating interference from carbohydrates [16,23,24]. The N-H bonds in threonine molecules exhibit characteristic absorption bands in the near-infrared spectral region. Detection of these N-H bond signals enables direct acquisition of threonine’s molecular fingerprint information. However, in grain samples, the O-H bonds from water molecules not only produce strong signals in the NIR region but also demonstrate significant spectral overlap with N-H bond absorption. Simultaneously, carbohydrates abundant in grains (containing C-H and O-H bonds) generate substantial background interference. It is particularly noteworthy that the CARS algorithm employed in this study successfully identified five key wavelengths from these complex spectral overlapping regions. These selected wavelengths effectively preserve the N-H characteristic response while eliminating major interfering factors such as carbohydrates, thereby enabling the developed model to specifically respond to variations in threonine content. The number of key wavelengths for the eight amino acids ranged from 3 to 7, accounting for 2.03% to 4.73% of the total spectral bands. Moreover, these wavelengths are consistently concentrated within the amino acid-sensitive region (characterized by O–H, C–H, and N–H vibrations) spanning 950–1650 nm [18]. This confirms the targeted screening capability of CARS in precisely locating biologically relevant features while eliminating irrelevant noise. This approach preserves characteristic wavelengths directly associated with amino acid functional groups while avoiding redundant information that dilutes effective signals. This is the core reason why the key wavelength model outperforms full-spectrum analysis.

### 4.4. Model Performance Determined by Alignment Between Regression Model Properties and Spectral Features

The core mechanistic differences among regression models define their compatibility with distinct spectral characteristics. PLSR models are based on a “linear relationship assumption” [19,20,21,35,36,37,48]. However, the spectral responses of amino acids in foxtail millet are influenced by intermolecular interactions (e.g., protein-carbohydrate binding), resulting in nonlinear characteristics. Hence, PLSR exhibited the poorest performance (all amino acids demonstrated R^2^ < 0.53 and RPD < 1.5). SVR employs kernel functions to map nonlinear data into a high-dimensional space, enabling the modeling of complex relationships [35,36,37,49,50]. For amino acids such as Thr, Val and His, which exhibit pronounced nonlinear spectral features, SVR effectively captures the implicit relationships between wavelengths. CNN extracts local features through convolutional and pooling layers, making it well-suited for amino acids with discrete and distinct characteristic peaks [34,35,36,37,51]. Taking Phe as an example, the CNN model can precisely target the strong localized peaks at 955 nm and 1280 nm, effectively filtering out interference from other wavelengths present in full-spectrum data. BiLSTM models continuous dependencies between wavelengths through its forward and backward temporal units. It is well-suited for amino acids with overlapping and continuous spectral features, such as the superimposed absorption peaks of amino and carboxyl groups in Lys. The key wavelengths of Lys (1172 nm, 1242 nm, 1252 nm, and 1388 nm) exhibit a continuous distribution, enabling BiLSTM to effectively learn the sequential dependencies and contextual patterns of their spectral responses [35,36,37,51].

### 4.5. Research Advantages, Limitations, and Future Prospects

This study pioneers the systematic comparison of four modeling approaches—PLSR, SVR, CNN, and BiLSTM—combined with CARS-based key wavelength screening for predicting eight essential amino acids in foxtail millet, providing a tailored predictive solution for each amino acid. CARS compresses the proportion of key wavelengths to 2.03–4.73%, significantly reducing the computational load of the models without compromising accuracy. Simultaneously, the accuracy of key wavelength models improved by approximately 5–20%, effectively balancing the dual demands of rapid detection and high precision. As a primary coarse cereal, the content of essential amino acids in foxtail millet serves as a core indicator for nutritional assessment. The models established in this study can replace traditional chemical detection methods, enabling real-time nutritional monitoring during foxtail millet processing and storage.

This study involved foxtail millet samples from a single variety and geographic origin, which may limit the model’s generalization capability. Only the CARS algorithm was employed for key wavelength selection, without cross-validation with other feature extraction methods, which may lead to a potential risk of over-screening. The lack of model integration may result in missed opportunities to leverage the synergistic advantages of combining localized features with temporal dependencies. The inferred correlations between amino acids were based on spectral data alone, without validation through metabolomics or molecular biology experiments.

Future work could involve collecting millet samples from diverse varieties, regions, and storage conditions to construct a multi-dimensional sample library, thereby enhancing model generalization through cross-validation and algorithm integration. The influence of processing parameters on amino acid spectral features can be explored to establish a full industrial chain predictive model, enabling quality control and nutritional optimization from raw material to finished products. The hybrid “CARS+” approach will be employed for joint screening of key wavelengths to mitigate over-screening risks. A hybrid model will be developed to adapt to the dual characteristics—localized and temporal features—of amino acid spectra. The attention mechanism is incorporated to enable the model to autonomously prioritize critical wavelengths. The portable near-infrared detection device will be developed based on the optimal models to enable on-site rapid testing during millet procurement and processing, thereby promoting technological implementation. The methodology will be extended to predict essential amino acids in other coarse grains, such as oats and buckwheat, establishing a universal NIR prediction framework for amino acids in coarse grains to provide a unified detection technology for the nutritional assessment of whole grains.

## 5. Conclusions

This study employed Changnong No. 47 foxtail millet as the research subject and established rapid non-destructive detection models for eight essential amino acids using near-infrared hyperspectral imaging based on 217 samples. The main conclusions are as follows:

First, spectral preprocessing and key wavelength extraction significantly improved data quality. Through S–G smoothing, airPLS baseline correction, and SNV transformation, noise and scattering interference were effectively suppressed. The CARS algorithm optimized and selected 3–7 key wavelengths, accounting for 2.03–4.73% of the full spectrum, which correspond to the absorption regions of characteristic functional groups (e.g., N–H, C–H) of the amino acids. This approach significantly reduced data dimensionality and enhanced model interpretability.

Second, the modeling strategy based on feature wavelengths generally outperformed full-spectrum models. The prediction accuracy—evaluated using R^2^, RMSE, and RPD—improved for all amino acids when key wavelengths were used. This indicates that the CARS effectively captures spectral information most relevant to amino acid content while reducing matrix interference.

Third, the optimal modeling approaches varied by amino acid. Lys was optimally modeled by BiLSTM. Phe, Met, Ile, and Leu were better suited to CNN. while SVR delivered the best performance for predicting Thr, Val, and His. All these methods significantly outperform the traditional PLSR approach.

This study provides an effective approach for rapid and non-destructive detection of amino acids in foxtail millet, overcoming the limitations of traditional methods such as sample destruction and complex procedures. The proposed methodology demonstrates broad application potential in quality breeding, process monitoring, and quality grading. Although the current models are based on a single variety, this work lays the foundation for future cross-variety generalization, development of portable devices, and quality analysis of other coarse grains. It holds significant theoretical value and practical potential.

## Figures and Tables

**Table 1:** Optimal key wavelengths screened by the CARS for eight essential amino acids in foxtail millet.

Type	NKW and PTW ^1^	Key Wavelengths (nm)
Lys	4/2.70%	1172, 1242, 1252, 1388
Phe	3/2.03%	955, 1280, 1398
Met	4/2.70%	1082, 1129, 1176, 1544
Thr	5/3.38%	1186, 1388, 1469, 1478, 1605
Ile	3/2.03%	1143, 1228, 1398
Leu	5/3.38%	997, 1134, 1186, 1242, 1318
Val	7/4.73%	988, 1101, 1134, 1139, 1242, 1346, 1643
His	6/4.05%	955, 969, 1176, 1223, 1412, 1431

^1^ NKW and PTW: The number of key wavelengths and their proportion in the total wavelength range.

## Data Availability

The original contributions presented in the study are included in the article, further inquiries can be directed to the corresponding author.

## Supplementary Materials

The following supporting information can be downloaded at: https://www.mdpi.com/article/10.3390/foods14213760/s1. Figure S1: Feature band extraction process for Lys using the CARS algorithm. Figure S2: Feature band extraction process for Phe using the CARS algorithm. Figure S3: Feature band extraction process for Met using the CARS algorithm. Figure S4: Feature band extraction process for Thr using the CARS algorithm. Figure S5: Feature band extraction process for Leu using the CARS algorithm. Figure S6: Feature band extraction process for Val using the CARS algorithm. Figure S7: Feature band extraction process for His using the CARS algorithm. Figure S8: Evaluation results of models established based on full-spectrum data for eight essential amino acids in foxtail millet.

## Abbreviations

The following abbreviations are used in this manuscript:

Lys	Lysine
Phe	Phenylalanine
Met	Methionine
Thr	Threonine
Ile	Isoleucine
Leu	Leucine
Val	Haline
His	Histidine
S–G	Savitzky–Golay
airPLS	Adaptive Iteratively Reweighted Penalized Least Squares
SNV	Standard Normal Variate
CARS	Competitive Adaptive Reweighted Sampling
PLSR	Partial Least Squares Regression
SVR	Support Vector Regression
CNN	Convolutional Neural Network
BiLSTM	Bidirectional Long Short-Term Memory
ROI	Regions of Interest
MCS	Monte Carlo Sampling
EDF	Exponentially Decreasing Function
ARS	Adaptive Reweighted Sampling
RMSECV	Root Mean Square Error of Cross–Validation
R^2^	Correlation coefficient
RMSE	Root mean square error
RPD	Residual prediction deviation
EAA	Essential Amino Acids
