# Supplementary material for: Rapid and Non-Destructive Assessment of Eight Essential Amino Acids in Foxtail Millet: Development of an Efficient and Accurate Detection Model Based on Near-Infrared Hyperspectral
Source: Foods. 2025 Nov 1;14(21):3760. doi: 10.3390/foods14213760 (PMC12607515; doi:10.3390/foods14213760)
Supplement: Supplementary file 1 [file foods-14-03760-s001.zip › foods-3923522-supplementary.pdf]

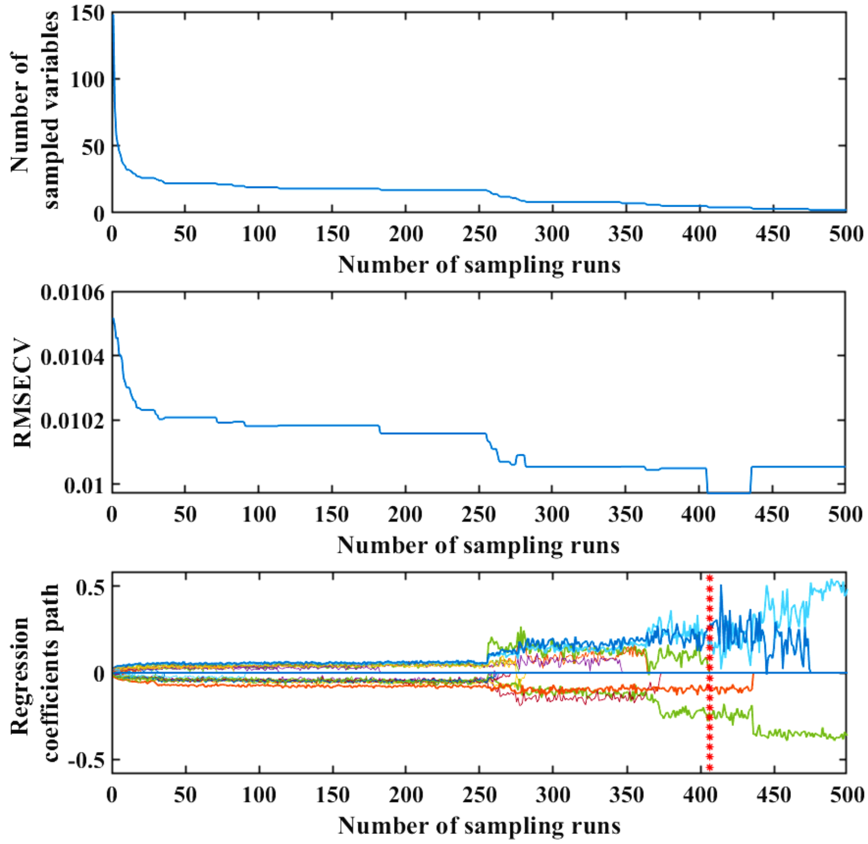

Figure S1 Feature band extraction process for *Lys* using the CARS algorithm.

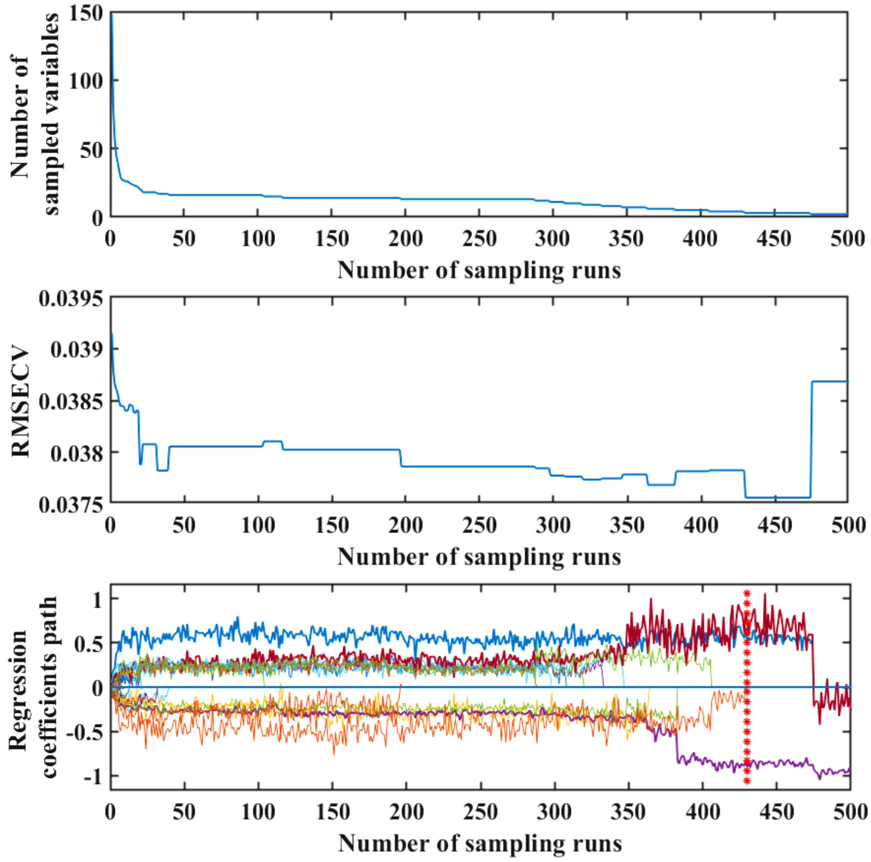

Figure S2 Feature band extraction process for *Phe* using the CARS algorithm.

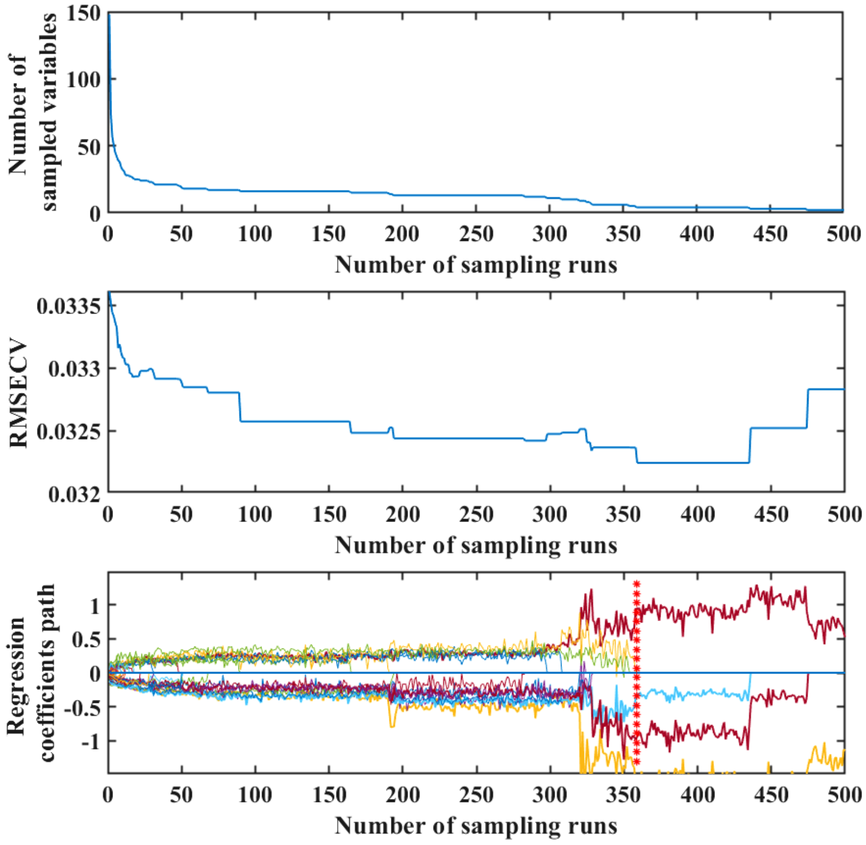

Figure S3 Feature band extraction process for *Met* using the CARS algorithm.

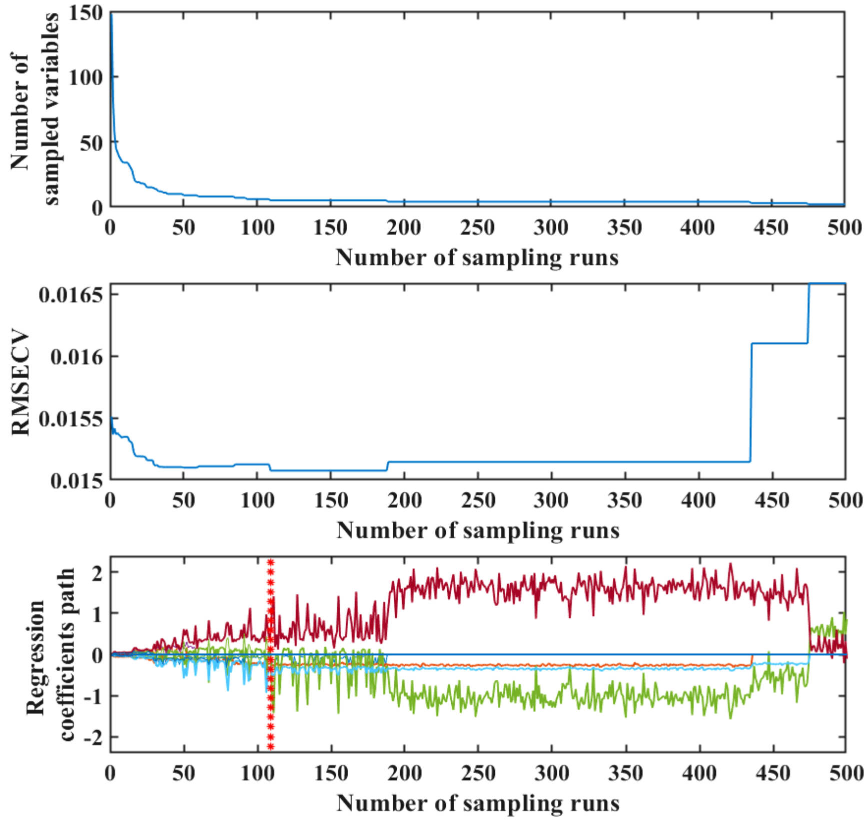

Figure S4 Feature band extraction process for *Thr* using the CARS algorithm.

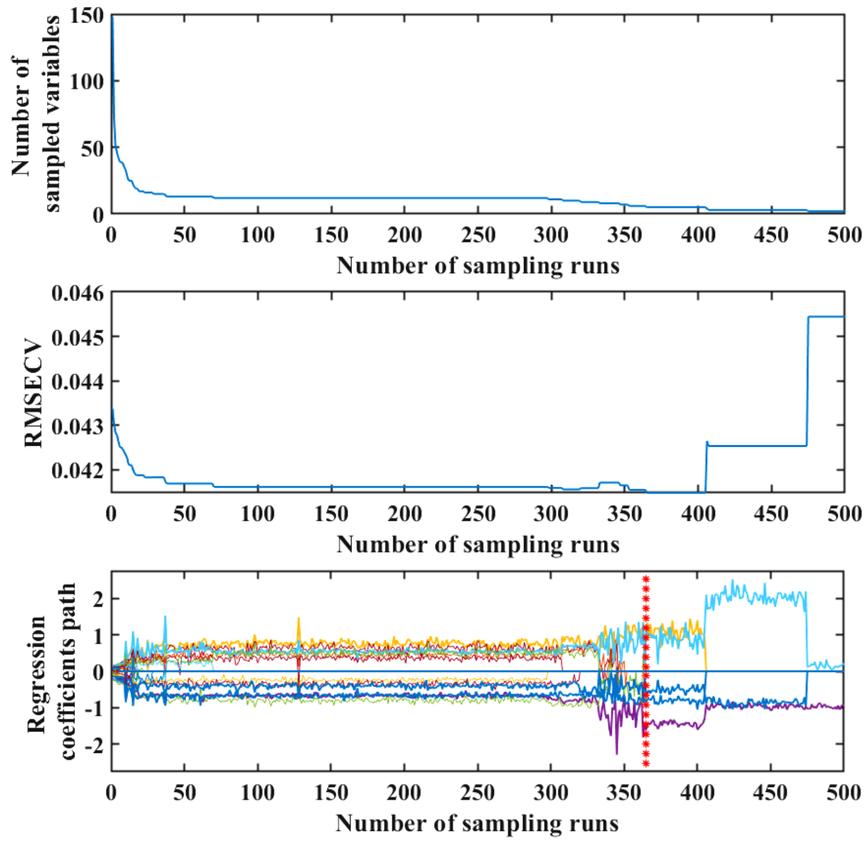

Figure S5 Feature band extraction process for *Leu* using the CARS algorithm.

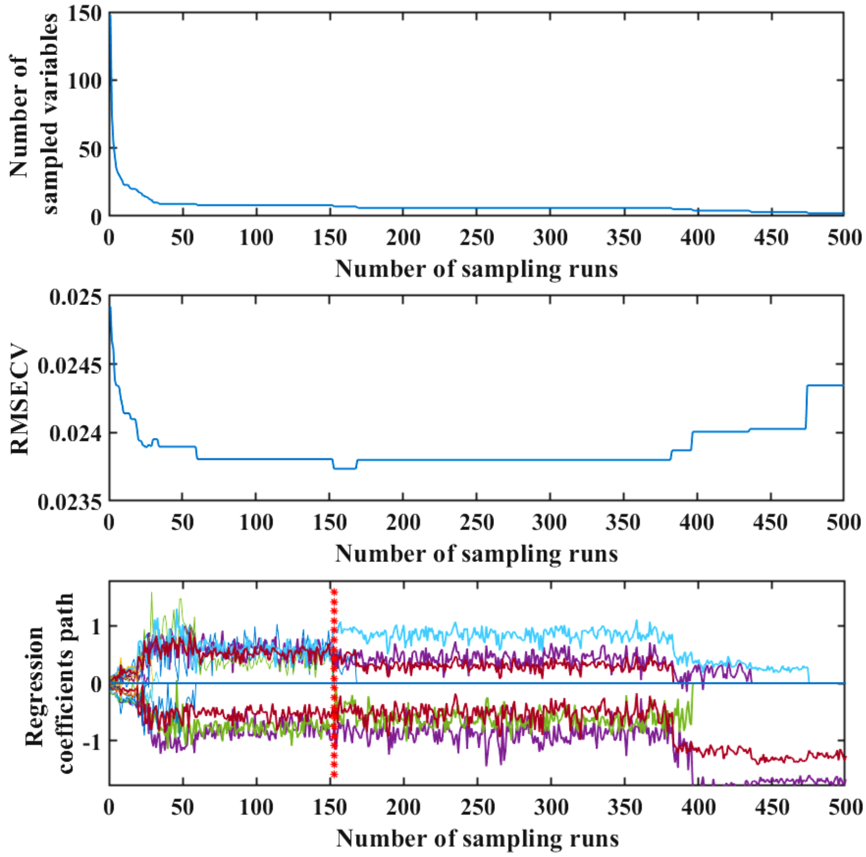

Figure S6 Feature band extraction process for *Val* using the CARS algorithm.

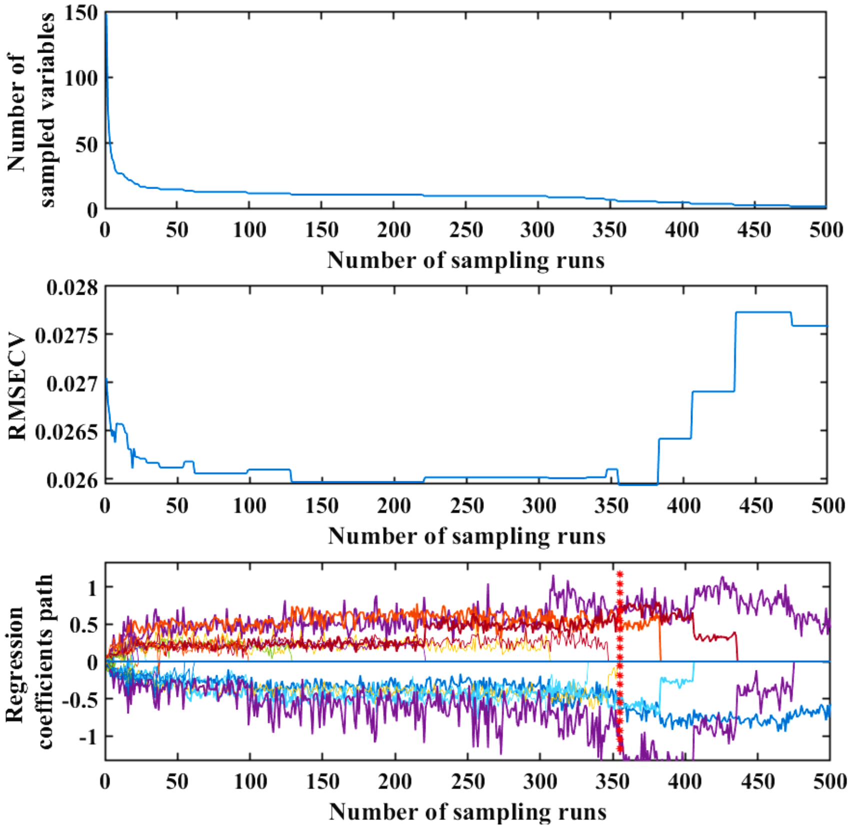

Figure S7 Feature band extraction process for *His* using the CARS algorithm.

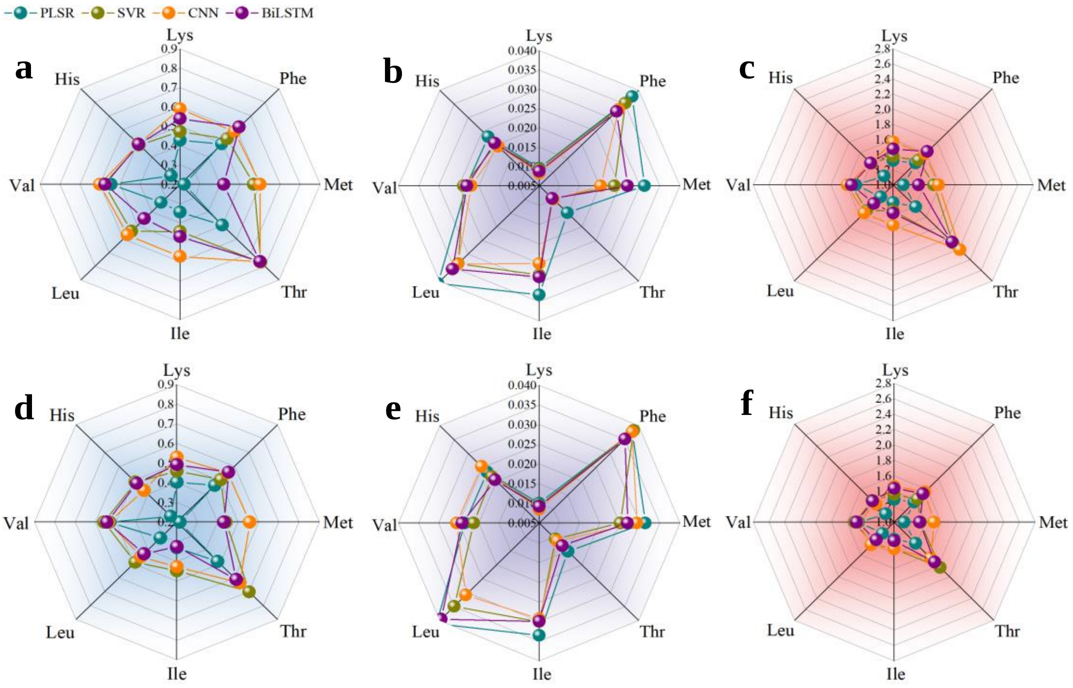

Figure S8 Evaluation results of models established based on full-spectrum data for eight essential amino acids in foxtail millet. (a) R^2 in the training set; (b) RMSE in the training set; (c) RPD in the training set; (d) R^2 in the prediction set; (e) RMSE in the prediction set; (f) RPD in the

prediction set.
